# Bioinformatics approach to identify the influences of SARS-COV2 infections on atherosclerosis

**DOI:** 10.3389/fcvm.2022.907665

**Published:** 2022-08-18

**Authors:** Jiuchang Zhang, Liming Zhang

**Affiliations:** Department of Neurology, The First Affiliated Hospital of Harbin Medical University, Harbin, China

**Keywords:** COVID-19, atherosclerosis, C1q, SARS-CoV-2, immune

## Abstract

Coronavirus disease (COVID-19), caused by severe acute respiratory syndrome coronavirus 2 (SARS-CoV-2), has been a global pandemic since early 2020. Understanding the relationship between various systemic disease and COVID-19 through disease ontology (DO) analysis, an approach based on disease similarity studies, has found that COVID-19 is most strongly associated with atherosclerosis. The study provides new insights for the common pathogenesis of COVID-19 and atherosclerosis by looking for common transcriptional features. Two datasets (GSE152418 and GSE100927) were downloaded from GEO database to search for common differentially expressed genes (DEGs) and shared pathways. A total of 34 DEGs were identified. Among them, ten hub genes with high degrees of connectivity were picked out, namely C1QA, C1QB, C1QC, CD163, SIGLEC1, APOE, MS4A4A, VSIG4, CCR1 and STAB1. This study suggests the critical role played by Complement and coagulation cascades in COVID-19 and atherosclerosis. Our findings underscore the importance of C1q in the pathogenesis of COVID-19 and atherosclerosis. Activation of the complement system can lead to endothelial dysfunction. The DEGs identified in this study provide new biomarkers and potential therapeutic targets for the prevention of atherosclerosis.

## Introduction

Coronavirus disease (COVID-19), caused by severe acute respiratory syndrome coronavirus 2 (SARS-CoV-2), has been a global pandemic since early 2020. According to the WHO, until March 9, 2022, the number of confirmed cases worldwide was 448,313,293, including 6,011,482 deaths (1). In response to the COVID-19 pandemic, a global effort is in progress to develop a vaccine against SARS-CoV-2. Vaccination can help control SARS-CoV-2 outbreaks by preventing infection, reducing disease severity, and blocking transmission (2). COVID-19 is typically characterized by upper respiratory symptoms, including fever, cough, and fatigue, and it is often accompanied by pulmonary infection (3). In addition to typical symptoms, some patients have serious cardiovascular damage, or even the first symptoms. (4) Except for the traditional established risk factors for atherosclerosis, such as age, smoking, hyperlipidemia, and hypertension, viral infection has been supposed to be a potential implication in atherosclerosis (5). SARS-CoV-2 binds to ACE2 to gain intracellular entry, leading to endothelial dysfunction (6). SARS-CoV-2 also promotes the accumulation of perivascular adipose tissue (7). These may exacerbate the underlying pathology of cardiovascular disease, leading to accelerated progression of atherosclerosis.

The purpose of this study was to explore the pathophysiological association between SARS-CoV-2 and atherosclerosis, and to better understand the underlying mechanisms, so as to facilitate early detection and prevention of atherosclerosis. Two gene expression datasets (GSE152418 and GSE100927) were downloaded from Gene Expression Omnibus (GEO) database. We used bioinformatics and enrichment analysis to determine the common DEGs and their functions for COVID-19 and atherosclerosis. In addition, protein protein interaction (PPI) networks were established to reveal hub genes. These data can better understand the potential link between the two diseases and provide evidence for therapeutic targets.

## Materials and methods

### Microarray data

The GSE152418 and GSE100927 gene expression profile were obtained from the GEO database (https://www.ncbi.nlm.nih.gov/geo) [Illumina NovaSeq 6000 (Homo sapiens)] platform was used for the GSE152418 dataset where samples were got from seventeen COVID-19 patients, and seventeen healthy people. On the contrary, for the GSE100927 dataset, GPL17077 [Agilent-039494 SurePrint G3 Human GE v2 8x60K Microarray 039381 (Probe Name version)] platform was adopted where samples were collected from sixty-nine atherosclerotic patients and thirty-five control subjects (Table 1).

**Table 1:** Basic information of the two microarray databases derived from the GEO database.

**Disease name**	**Dataset ID**	**Subjects**	**GEO platform**	**Number of samples(control/disease)**
COVID-19	GSE152418	Peripheral blood mononuclear cell	GPL24676	17/17
Atherosclerosis	GSE100927	Carotid, femoral and infra-popliteal arteries	GPL17077	35/69

*GEO, Gene Expression Omnibus; COVID-19, Coron a Virus Disease 2019*.

### Disease ontology (DO) analysis

Firstly, the “edgeR” package was applied to screen Differentially Expressed Genes (DEGs) from the GSE152418 dataset and a adjusted *P* less than 0.05, and |log2 Fold change (FC)| more than or equal to 1 was set as a cut-off point for selecting DEGs. The “DOSE” (8). and “ClusterProfiler” (9). packages were then used for DO analysis to study the disease mechanism by looking for disease correlation. DO analysis is a method based on the study of disease similarity, and it plays a vital role in understanding the pathogenesis of complex diseases, the early prevention and diagnosis of major diseases, new drug development, and drug safety evaluation.

### Acquisition of common genes

The LIMMA package was used to detect the DEGs between atherosclerotic patients and healthy control from the GSE100927 dataset, and the adjusted P-value and |log2FC| were calculated. Genes that met the cutoff criteria, adjusted *P* < 0.05 and |log2FC| more than or equal to 1.0, were considered as DEGs. Then the common genes of the GSE152418 and GSE100927 sets were identified by using the Venn diagram webtool (bioinformatics.psb.ugent.be/webtools/Venn/).

### Enrichment analysis of common genes

To further analyze biological processes of common DEGs, GO annotation analysis and KEGG pathway enrichment analysis were carried out through the Database for Annotation, Visualization and Integrated Discovery [DAVID (2021 Update), https://david.ncifcrf.gov/]. *P*-Value < 0.05 was used as the enrichment screening condition.

### Construction PPI network and selection hub genes

The PPI network was predicted using Search Tool for the Retrieval of Interacting Genes (STRING, version 11.5, http://string-db.org/) online database. The PPI pairs were extracted with a interaction score more than or equal to 0.15, and then the PPI network was visualized by Cytoscape software (www.cytoscape.org/). Here, we used Degree to evaluate and select hub genes.

## Results

### DO analysis

**Figure 1:**
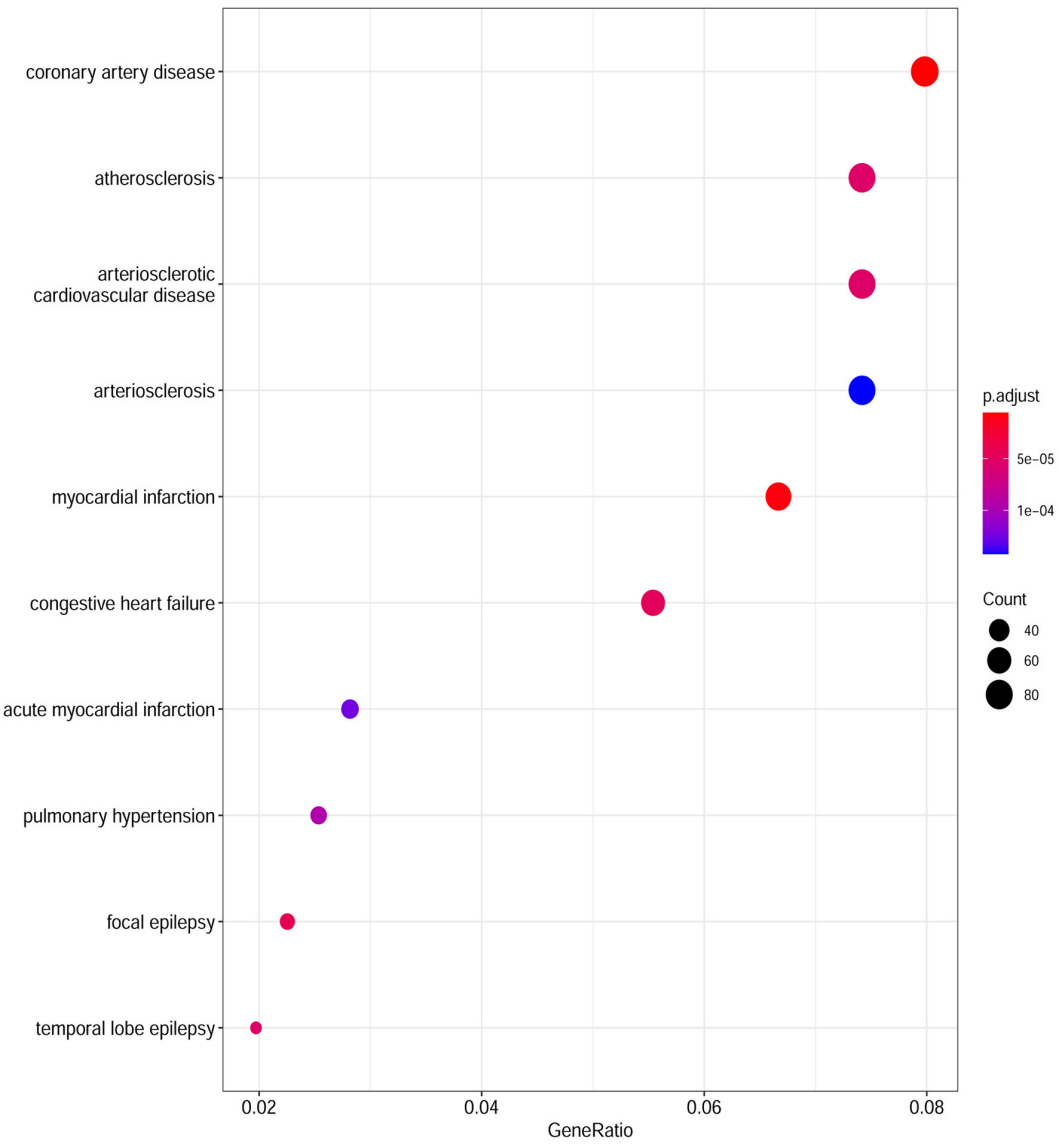
Disease Ontology (DO) analysis of the DEGs from the GSE152418 dataset. The size of the circle represents the number of genes involved, and the abscissa represents the frequency of the genes involved in the term total genes.

Based on the cut-off criteria of adjusted *P* < 0.05 and |log2FC| more than or equal to 1, a total of 2080 DEGs were identified from GSE152418, including 1905 upregulated genes and 175 downregulated genes. p.adjust < 0.05 and gene counts more than or equal to 20 were used as the DO screening condition. Figure 1 shows the top ten most significantly enriched diseases, coronary artery disease, atherosclerosis, arteriosclerotic cardiovascular disease, arteriosclerosis, myocardial infarction, congestive heart failure, acute myocardial infarction, pulmonary hypertension, focal epilepsy and temporal lobe epilepsy. The enrichment results of other diseases by DO analysis are shown in Table 2.

**Table 2:** Significantly enriched DO terms of DEGs.

**DO ID**	**Description**	**Count**	***P*-Value**	***P*-Adjust**
DOID:3393	Coronary artery disease	85	6.97E−09	5.83E−06
DOID:5844	Myocardial infarction	71	2.17E−08	9.07E−06
DOID:2234	Focal epilepsy	24	1.52E−07	4.24E−05
DOID:6000	Congestive heart failure	59	2.20E−07	4.59E−05
DOID:3328	Temporal lobe epilepsy	21	3.18E−07	5.32E−05
DOID:1936	Atherosclerosis	79	3.97E−07	5.38E−05
DOID:2348	Arteriosclerotic cardiovascular disease	79	4.50E−07	5.38E−05
DOID:6432	Pulmonary hypertension	27	9.09E−07	9.50E−05
DOID:9408	Acute myocardial infarction	30	1.34E−06	0.000124898
DOID:2349	Arteriosclerosis	79	1.70E−06	0.000142212
DOID:5679	Retinal disease	77	7.92E−06	0.000601666
DOID:1168	Familial hyperlipidemia	26	1.20E−05	0.000834386
DOID:3146	Lipid metabolism disorder	28	1.34E−05	0.00085861
DOID:1793	Pancreatic cancer	69	1.94E−05	0.00115929
DOID:850	Lung disease	98	2.75E−05	0.001532539
DOID:4450	Renal cell carcinoma	72	3.34E−05	0.001746893
DOID:8466	Retinal degeneration	58	3.98E−05	0.001958846
DOID:6364	Migraine	23	4.23E−05	0.00196426
DOID:3324	Mood disorder	45	5.17E−05	0.00227529
DOID:0080000	Muscular disease	82	5.54E−05	0.002316532
DOID:263	Kidney cancer	86	8.04E−05	0.003053437
DOID:3459	Breast carcinoma	77	9.26E−05	0.003364892
DOID:0060037	Developmental disorder of mental health	75	0.000113602	0.003794572
DOID:1826	Epilepsy syndrome	46	0.000118977	0.003794572
DOID:4451	Renal carcinoma	76	0.000122552	0.003794572
DOID:1686	Glaucoma	29	0.00014554	0.004217764
DOID:2355	Anemia	53	0.0001587	0.004416723
DOID:2742	Auditory system disease	27	0.000187263	0.00470284
DOID:936	Brain disease	87	0.000192221	0.00470284
DOID:15	Reproductive system disease	76	0.000205883	0.00470284
DOID:120	Female reproductive organ cancer	87	0.000207772	0.00470284
DOID:74	Hematopoietic system disease	90	0.00020814	0.00470284
DOID:3996	Urinary system cancer	94	0.000217288	0.00473576
DOID:4074	Pancreas adenocarcinoma	38	0.000234928	0.00473576
DOID:0060040	Pervasive developmental disorder	45	0.000239673	0.00473576
DOID:0060116	Sensory system cancer	34	0.000241385	0.00473576
DOID:2174	Ocular cancer	34	0.000241385	0.00473576
DOID:0060041	Autism spectrum disorder	43	0.000254915	0.00473576
DOID:12849	Autistic disorder	43	0.000254915	0.00473576
DOID:18	Urinary system disease	90	0.000280864	0.005097229
DOID:3083	Chronic obstructive pulmonary disease	48	0.000286567	0.005097229
DOID:423	Myopathy	77	0.00033099	0.005647089
DOID:66	Muscle tissue disease	77	0.00033099	0.005647089
DOID:374	Nutrition disease	67	0.000399054	0.006672186
DOID:0050700	Cardiomyopathy	35	0.000449318	0.007240401
DOID:6713	Cerebrovascular disease	29	0.000459021	0.007240401
DOID:654	Overnutrition	64	0.000496562	0.007574304
DOID:4905	Pancreatic carcinoma	50	0.000498309	0.007574304
DOID:557	Kidney disease	86	0.000522392	0.007798559
DOID:4645	Retinal cancer	29	0.000620866	0.008650729
DOID:9970	Obesity	62	0.000663498	0.008946528
DOID:1115	Sarcoma	42	0.000711231	0.009147531
DOID:229	Female reproductive system disease	42	0.000711231	0.009147531
DOID:2320	Obstructive lung disease	61	0.000734442	0.009302929
DOID:10534	Stomach cancer	55	0.000950468	0.011515815
DOID:768	Retinoblastoma	28	0.001033977	0.012138426
DOID:771	Retinal cell cancer	28	0.001033977	0.012138426
DOID:3312	Bipolar disorder	35	0.001091638	0.012501503
DOID:9352	Type 2 diabetes mellitus	45	0.001110794	0.012548966
DOID:5041	Esophageal cancer	33	0.001158125	0.012909231
DOID:3770	Pulmonary fibrosis	29	0.001261354	0.013519123
DOID:403	Mouth disease	40	0.001484673	0.015136423
DOID:26	Pancreas disease	37	0.001605415	0.015977706
DOID:633	Myositis	21	0.001648237	0.016210895
DOID:0060085	Organ system benign neoplasm	52	0.00167946	0.01632591
DOID:4607	Biliary tract cancer	38	0.001807765	0.017371169
DOID:0060084	Cell type benign neoplasm	85	0.002061029	0.019359779
DOID:657	Adenoma	64	0.002109774	0.019597453
DOID:1074	Kidney failure	34	0.002145424	0.019709612
DOID:48	Male reproductive system disease	30	0.002200606	0.019996808
DOID:865	Vasculitis	28	0.002325529	0.020904752
DOID:8398	Osteoarthritis	39	0.002473537	0.021998689
DOID:0060100	Musculoskeletal system cancer	79	0.002508247	0.022023954
DOID:299	Adenocarcinoma	34	0.002676763	0.022177269
DOID:5223	Infertility	42	0.002695257	0.022177269
DOID:3082	Interstitial lung disease	36	0.00271993	0.022177269
DOID:1575	Rheumatic disease	39	0.002732367	0.022177269
DOID:418	Systemic scleroderma	39	0.002732367	0.022177269
DOID:419	Scleroderma	39	0.002732367	0.022177269
DOID:2394	Ovarian cancer	59	0.002780859	0.022331756
DOID:201	Connective tissue cancer	68	0.002894952	0.022331756
DOID:10952	Nephritis	32	0.002911676	0.022331756
DOID:3620	Central nervous system cancer	28	0.002988188	0.022505631
DOID:0080015	Physical disorder	30	0.003147388	0.023285098
DOID:4960	Bone marrow cancer	61	0.003537522	0.025494557
DOID:0070004	Myeloma	60	0.003905917	0.026264796
DOID:2621	Autonomic nervous system neoplasm	68	0.004049339	0.026264796
DOID:769	Neuroblastoma	68	0.004049339	0.026264796
DOID:1091	Tooth disease	34	0.004084239	0.026264796
DOID:10825	Essential hypertension	27	0.004708132	0.028812356
DOID:289	Endometriosis	21	0.00483272	0.029276479
DOID:854	Collagen disease	40	0.005216331	0.03128014
DOID:1107	Esophageal carcinoma	27	0.005290025	0.031364972
DOID:0050737	Autosomal recessive disease	61	0.005365584	0.031588932
DOID:0060036	Intrinsic cardiomyopathy	29	0.00544447	0.03160817
DOID:127	Leiomyoma	22	0.005828457	0.032922905
DOID:37	Skin disease	63	0.00656053	0.035847078
DOID:1192	Peripheral nervous system neoplasm	70	0.006679809	0.036261818
DOID:552	Pneumonia	24	0.007057917	0.037823198
DOID:4766	Embryoma	63	0.007450764	0.039423029
DOID:3388	Periodontal disease	29	0.008300314	0.04322301
DOID:16	Integumentary system disease	69	0.008758512	0.044109131
DOID:0060038	Specific developmental disorder	42	0.009006264	0.044816886
DOID:12930	Dilated cardiomyopathy	21	0.009380129	0.04640111
DOID:230	Lateral sclerosis	24	0.009987727	0.047987012

*DEG, Differentially Expressed Gene; DO, Disease Ontology; ID, Identity Document*.

### Identification of common DEGs

**Figure 2:**
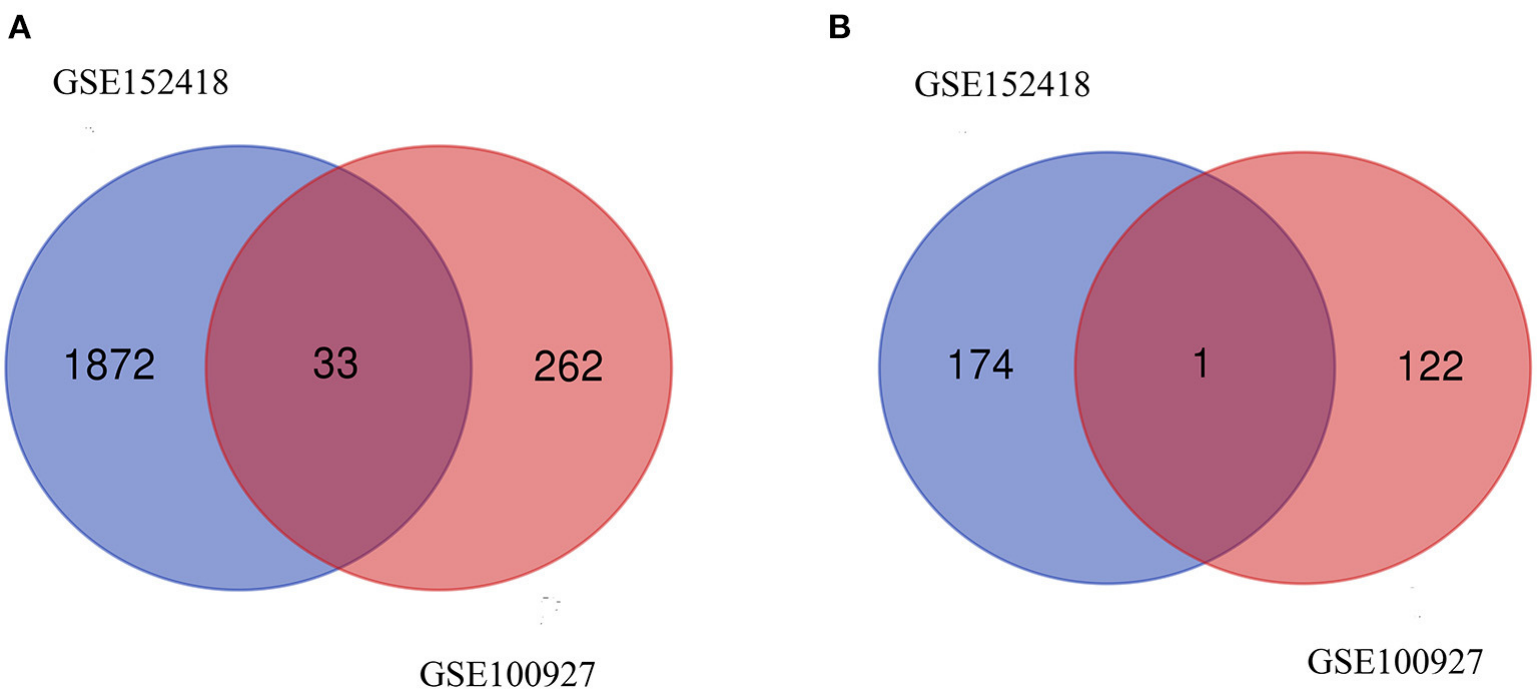
Venn diagram. **(A)** Upregulated common DEGs of the GSE152418 and GSE100927 datasets. **(B)** Downregulated common DEGs of the GSE152418 and GSE100927 datasets.

From GSE100927, 418 DEGs including 295 upregulated genes and 123 downregulated genes were identified. We analyzed the intersection of the DEG profiles using Venn (Figure 2). Ultimately, 34 DEGs were significantly differentially expressed in two datasets, of which 33 were significantly upregulated genes and 1 was downregulated gene.

### Gene ontology and pathway enrichment analysis

GO and KEGG pathway analyses for DEGs were performed using the DAVID. The biological processes of DEGs were primarily associated with synapse disassembly, complement activation and innate immune response. For the cell component, the DEGs were enriched in extracellular region, blood microparticle, hemoglobin complex, collagen trimer, and so on. Molecular functions analysis showed that the DEGs were significantly enriched in oxygen transporter activity, oxygen binding, scavenger receptor activity, voltagE–gated potassium channel activity involved in atrial cardiac muscle cell action potential repolarization, phosphatidylcholinE–sterol O-acyltransferase activator activity, haptoglobin binding, organic acid binding and heme binding (Table 3). In addition, the KEGG pathway analysis showed that the DEGs were significantly enriched in Complement and coagulation cascades, Pertussis, Coronavirus disease—COVID-19, Staphylococcus aureus infection, Chagas disease, Systemic lupus erythematosus and Alcoholic liver disease (Table 4).

**Table 3:** Significantly enriched GO terms of DEGs.

**GO ID**	**Description**	**Count**	***P*-Value**	**Genes**
**Biological process**				
GO:0098883	Synapse disassembly	3	6.04E−05	C1QB, C1QA, C1QC
GO:0006958	Complement activation, classical pathway	5	8.67E−05	C1QB, C1QA, IGLL5, IGLL1, C1QC
GO:0045087	Innate immune response	7	2.32E−04	C1QB, C1QA, IGLL5, VNN1, IGLL1, C1QC, OASL
GO:0006898	Receptor-mediated endocytosis	4	0.001825851	CD163, STAB1, HBA2, APOE
GO:0006954	Inflammatory response	5	0.002915761	CCR1, VNN1, STAB1, SPP1, SIGLEC1
GO:0098869	Cellular oxidant detoxification	3	0.00591978	HBA2, HBD, APOE
GO:0042159	Lipoprotein catabolic process	2	0.007472267	APOE, CTSD
GO:0098914	Membrane repolarization during atrial cardiac muscle cell action potential	2	0.007472267	KCNJ5, KCNA5
GO:0006956	Complement activation	3	0.008884327	C1QB, C1QA, C1QC
GO:0034447	Very-low-density lipoprotein particle clearance	2	0.008960238	APOC1, APOE
GO:0034382	Chylomicron remnant clearance	2	0.010446055	APOC1, APOE
GO:0030449	Regulation of complement activation	3	0.012175792	C1QB, C1QA, C1QC
GO:0010873	Positive regulation of cholesterol esterification	2	0.013411239	APOC1, APOE
GO:0033700	Phospholipid efflux	2	0.017842935	APOC1, APOE
GO:0044267	Cellular protein metabolic process	3	0.021136392	MMP1, SPP1, APOE
GO:0015671	Oxygen transport	2	0.022255408	HBA2, HBD
GO:0015909	Long-chain fatty acid transport	2	0.025186416	FABP5, APOE
GO:0034375	High-density lipoprotein particle remodeling	2	0.026648738	APOC1, APOE
GO:0042157	Lipoprotein metabolic process	2	0.032476871	APOC1, APOE
GO:0033344	Cholesterol efflux	2	0.036825845	APOC1, APOE
GO:0045671	Negative regulation of osteoclast differentiation	2	0.039714668	MAFB, LILRB4
GO:0032703	Negative regulation of interleukin-2 production	2	0.041155941	VSIG4, LILRB4
GO:0042744	Hydrogen peroxide catabolic process	2	0.041155941	HBA2, HBD
GO:0010033	Response to organic substance	2	0.042595125	AQP9, KCNA5
GO:0007267	Cell-cell signaling	3	0.045927718	CCR1, C1QA, STAB1
GO:0042742	Defense response to bacterium	3	0.046288292	IGLL5, IGLL1, STAB1
**Cellular component**				
GO:0005576	Extracellular region	17	2.40E−08	C1QB, C1QA, CD163, CD163L1, MMP1, HBA2, VNN1, FNDC1, FABP5, IGLL1, APOC1, SPP1, PLBD1, SIGLEC1, APOE, CTSD, C1QC
GO:0072562	Blood microparticle	5	7.68E−05	C1QB, HBA2, HBD, APOE, C1QC
GO:0005833	Hemoglobin complex	3	2.19E−04	HBA2, HBD
GO:0005581	Collagen trimer	4	4.28E−04	C1QB, C1QA, MMP1, C1QC
GO:0009897	External side of plasma membrane	6	6.43E−04	CCR1, KCNJ5, IGLL5, CD163, CD163L1, IGLL1
GO:0005602	Complement component C1 complex	2	0.003171533	C1QB, C1QA
GO:0098794	Postsynapse	3	0.012921039	C1QB, C1QA, C1QC
GO:0031838	Haptoglobin-hemoglobin complex	2	0.017323254	HBA2, HBD
GO:0042627	Chylomicron	2	0.021997095	APOC1, APOE
GO:0071682	Endocytic vesicle lumen	2	0.028195396	HBA2, APOE
GO:0016021	Integral component of membrane	15	0.028427769	PTCRA, CCR1, KCNJ5, CD163, CD163L1, AQP9, KCNA5, HBD, LILRB4, MS4A4A, VNN1, SLCO2B1, STAB1, SIGLEC1, VSIG4
GO:0034361	Very-low-density lipoprotein particle	2	0.03281913	APOC1, APOE
GO:0045202	Synapse	4	0.041387143	C1QB, C1QA, FABP5, C1QC
GO:0034364	High-density lipoprotein particle	2	0.042002749	APOC1, APOE
**Molecular function**				
GO:0005344	Oxygen transporter activity	3	3.12E−04	HBA2, HBD
GO:0019825	Oxygen binding	3	0.001605913	HBA2, HBD
GO:0005044	Scavenger receptor activity	3	0.002957949	CD163, CD163L1, STAB1
GO:0086089	Voltage–gated potassium channel activity involved in atrial cardiac muscle cell action potential repolarization	2	0.006590464	KCNJ5, KCNA5
GO:0060228	Phosphatidylcholine–sterol O-acyltransferase activator activity	2	0.009869914	APOC1, APOE
GO:0031720	Haptoglobin binding	2	0.016397412	HBA2, HBD
GO:0043177	Organic acid binding	2	0.018022768	HBA2, HBD
GO:0020037	Heme binding	3	0.025666884	HBA2, HBD

*DEG, Differentially Expressed Gene; GO, Gene Ontology; ID, Identity Document*.

**Table 4:** Significantly enriched KEGG terms of DEGs.

**KEGG ID**	**Description**	**Count**	***P*-Value**	**Genes**
hsa04610	Complement and coagulation cascades	4	0.001112855	C1QB, C1QA, VSIG4, C1QC
hsa05133	Pertussis	3	0.014757214	C1QB, C1QA, C1QC
hsa05171	Coronavirus disease—COVID-19	4	0.018374812	C1QB, C1QA, MMP1, C1QC
hsa05150	Staphylococcus aureus infection	3	0.022928086	C1QB, C1QA, C1QC
hsa05142	Chagas disease	3	0.02567277	C1QB, C1QA, C1QC
hsa05322	Systemic lupus erythematosus	3	0.043532485	C1QB, C1QA, C1QC
hsa04936	Alcoholic liver disease	3	0.047059029	C1QB, C1QA, C1QC

*KEGG, Kyoto Encyclopedia of Genes and Genomes; ID, Identity Document; DEG, Differentially Expressed Gene*.

### PPI network construction and hub gene identification

**Figure 3:**
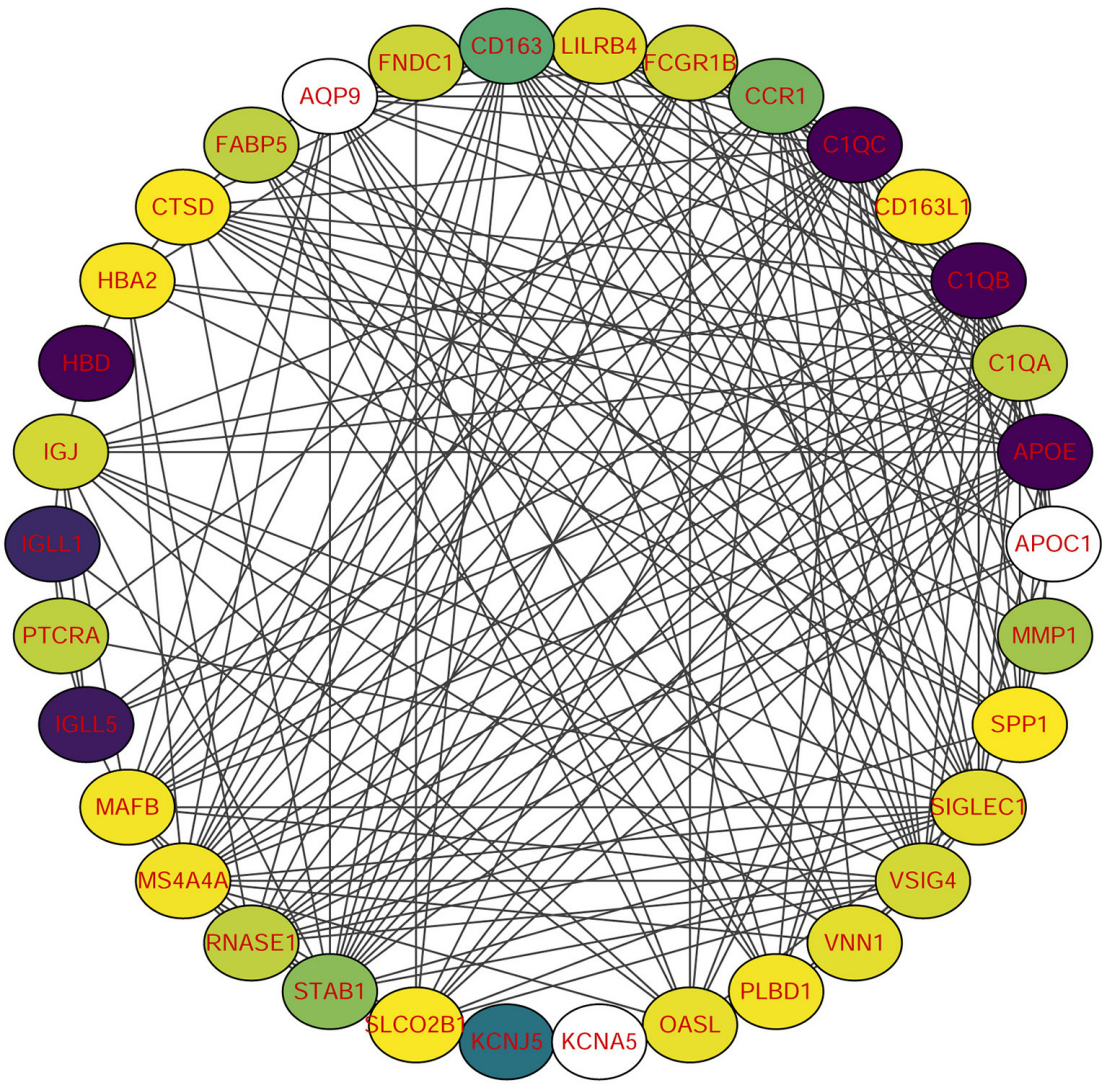
Protein-protein interaction (PPI) network of common DEGs among SRAS-CoV-2 and atherosclerosis. In the figure, the circle nodes represent DEGs and edges represent interactions between nodes.

Using STRING tools, we predicted protein interactions among DEGs. The PPI network presented in Figure 3 consists of 34 nodes and 209 edges. Based on the PPI network, we identified 10 genes with the highest connectivity degree (Table 5). The results showed that C1QA was the most outstanding gene with connectivity degree = 24, followed by C1QB (degree = 23), C1QC (degree = 22), CD163 (degree = 22), SIGLEC1 (degree = 21), APOE (degree = 19), MS4A4A (degree = 19), VSIG4 (degree = 18), CCR1 (degree = 18), STAB1 (degree = 18).

**Table 5:** Top ten hub genes with higher degree of connectivity.

**Gene symbol**	**Gene description**	**Degree**
C1QA	Complement C1q A chain	24
C1QB	Complement C1q B chain	23
C1QC	Complement C1q C chain	22
CD163	CD163 molecule	22
SIGLEC1	Sialic acid binding Ig like lectin 1	21
APOE	Apolipoprotein E	19
MS4A4A	Membrane spanning 4-domains A4A	19
VSIG4	V-set and immunoglobulin domain containing 4	18
CCR1	C-C motif chemokine receptor 1	18
STAB1	Stabilin 1	18

## Discussion

Some diseases, thought to be unrelated, share the same biological processes (10). We conducted the DO analysis on the GSE152418 dataset to find the similarity between diseases and COVID-19, and found that COVID-19 was most significantly associated with atherosclerosis among various diseases. Our results suggest that COVID-19 will lead to faster atherosclerosis. Then, we took the intersection of two datasets, GSE152418 and GSE100927, to identify common genes between COVID-19 and atherosclerosis. After obtaining 34 common genes, the GO, pathway, PPI networks were further analyzed.

GO enrichment analysis showed that C1QA, C1QB, C1QC were significantly enriched in synapse disassembly, complement activation, and innate immune response. Complement 1q (C1q) is composed of six subunits, which form a molecule containing 18 polypeptide chains, while C1qA, C1qB, and C1qC genes encode three types of polypeptide chains, A, B, and C of the subunit of C1q, respectively (11). C1q is an important recognition molecule to initiate the classical pathway involved in the complement activation and function, playing a major role in the connection between innate and specific immunity. (12, 13). After identifying the complement binding site on the antibody Fc segment of the IgM or IgG immune complex, the complement cascade will be activated to clear the antigen-antibody complexes (14). Complement proteins specifically locate apoptotic, immature or weak developing synapses in the central nervous system (15). The number of those apoptotic markers in the synapse is equal to the localization of C1q, which promotes synaptic pruning (16). A study found that of 281 patients diagnosed with COVID-19, 21.1% had dementia and 8.9% had mild cognitive impairment (MCI) (17). Moreover, high activation of C1q leads to a large number of synaptic loss which is associated with the development of Alzheimer's disease (18). Then, does the activation of complement system C1q cause cognitive impairment in COVID-19 patients?

KEGG enrichment analysis is the best way to reflect the changes of pathways in organisms. Those results indicate that complement and coagulation cascades change most significantly in atherosclerosis and COVID-19. Macor et al. found positive lung C1q staining which suggests that the classical pathway is important for complement activation which may be triggered by IgG, antibodies widely distributed in patients' lungs (19). In atherosclerosis plaques, C1q activates the classical complement pathway by recognizing oxidized low-density lipoprotein auto-antibodies or directly binding modified lipoprotein and cholesterol crystal (20). Endothelial dysfunction, an important mechanism for the formation and development of atherosclerosis, can be caused by the activation of the complement system can lead to (20). Gao et al. demonstrated that subsequent endothelial dysfunction persisted in COVID-19 survivors even 327 days after diagnosis (6). The activated fragments generated after the activation of the complement system may be closely related to the coagulation and fibrinolytic system and inflammation in COVID-19 patients, so additional studies on the changes in the number of fragments and tissue distribution are needed.

The 10 hub genes selected by PPI were C1QA, C1QB, C1QC, CD163, SIGLEC1, APOE, MS4A4A, VSIG4, CCR1, and STAB1. The C1QA, C1QB, and C1QC genes had the highest degree in the PPI networks. Then v-set and immunoglobulin domain containing 4 (VSIG4) is the receptor of complement component 3 fragments C3b and iC3b, which activates macrophage immunity through C3b/iC3b binding (21). VSIG4 may be involved in lung injury through induction of phagocytosis (22). VSIG4 activate macrophages, through induction of chemokines, promote the migration of inflammatory cells to the lesion area, and participate in the pathogenesis of arteriosclerosis (23). Increased expressions of C1QA, C1QB, C1QC, and VSIG4 all relate to enhanced complement system. CD163, a scavenger receptor, is a major component of inflammation and the immune response. Among plasmacytoid dendritic cells, type I interferon is induced with the appearance of CD163+ SIGLEC1+ macrophages with increased angiotensin converting enzyme 2 (ACE2) levels (24). Macrophages are highly enriched in the lungs of macaques at peak viremia and harbor the SARS-CoV-2 virus while also expressing an interferon-driven innate antiviral gene signature (25). CD163(+) macrophages promote angiogenesis, vascular permeability and inflammation in atherosclerosis via the CD163/HIF1α/VEGF-A pathway. The increased expression of CD163 was revealed in ruptured coronary plaques (26). There are three APOE isoforms, namely APOE epsilon2 (APOE2), APOE epsilon3 (APOE3) and APOE epsilon4 (APOE4) located on chromosome 19q13.2 (27). APOE can function as an endogenous, concentration-dependent pulmonary danger signal that primes and activates the NLPR3 inflammasome in bronchoalveolar lavage fluid macrophages from asthmatic subjects to secrete IL-1β (28). A recent study in the UK Biobank Cohort, APOE4 has been shown to associate with increased susceptibility to SARS-CoV-2 infection and COVID-19 mortality (29). APOE is a therapeutic target for statins that inhibit inflammation in patients with atherosclerotic vascular disease.

Statins possess antiviral, immunomodulatory, antithrombotic, and anti-inflammatory properties, which may improve short- and long-term outcomes in COVID-19 patients.

STAB1 encodes an unusual type of multifunctional scavenger receptor that causes increased lipid uptake and transient lipid depletion in virus-infected areas and is associated with poor prognosis for COVID-19 (30). STAB1 expression may contribute to foam cell formation, monocyte adhesion/migration, and regulation of inflammation in atherosclerotic lesions (31). Lectins such as sialic acid-binding Ig-like lectin 1 (SIGLEC1/CD169) mediate the attachment of viruses to Antigen-presenting cells (APCs) (32). SIGLEC1 expression is induced on APCs upon IFN-α or LPS exposure and increased in myeloid cells of COVID-19 patients (33) Inhibition of Siglec-1 prevents monocytes from adhering to vascular endothelial cells in the early stage of atherosclerosis, and reduces lipid phagocytosis and chemokine secretion of macrophages, alleviating the inflammatory response of established fat streaking lesions (34). CCR1 is critical mediators of monocyte/macrophage polarization and tissue infiltration, which are pathogenic hallmarks of severe COVID-19 (35). The use of monocyte CCR1 in arterial recruitment is due in part to activated chemokines of platelet deposition, which is important in the early stages of atherosclerosis (36). MS4A4A is a novel M2 macrophage cell surface marker, which is essential for dectin-1-dependent activation of NK cell-mediated anti-metastatic properties (37). Silva-Gomes et al. found MS4A4A was expressed by MΦs or alveolar MΦs in COVID-19 bronchoalveolar lavage fluid (38).

Through DO analysis, we also found several neurological disorders associated with COVID-19, such as focal epilepsy, temporal lobe epilepsy, migraine, epilepsy syndrome, neuroblastoma, and lateral sclerosis. There have been a large number of reported cases of these conditions, with a seizure prevalence ranging from 0 to 26% in COVID-19 patients (39, 40). Moreover, seizures may be related to cerebrovascular disease and central nervous system infection. Vascular endothelial injury leads to hypercoagulability and microembolism, resulting in reduced cortical blood flow accompanied by hypoxia. Vascular endothelial dysfunction can lead to changes in the nervous system, resulting in neurological sequelae (41). The Atherosclerosis Risk in Communities (ARIC) study also revealed that migraine patients were more susceptible to retinopathy (retinal hemorrhage, macular oedema, retinal microvascular abnormalities, venous bleeding, etc.) than non-migraine patients, and retinopathy was more strongly associated with migraine in people without a history of diabetes or hypertension (42). Interestingly, we also discovered DEGs enrichment in retinopathy. Besides, previous animal-based experimental studies of the coronavirus infection reported retinal diseases such as retinal vasculitis and retinal degeneration. Moreover, blood-retinal barrier breakdown revealed the possibility of immune-privileged site infectivity by SARS-CoV-2 (43). We believe that SARS-CoV-2 causes vascular injury and may lead to retinal degeneration. Results also revealed different types of cancer, such as pancreatic cancer, kidney cancer, breast carcinoma, stomach cancer, esophageal cancer, and ovarian cancer. In patients with COVID19, severe illness and mortality are closely related to cancer. SARS CoV 2 may promote tumor progression and stimulate metabolic switching in tumor cells to initiate tumor metabolic modes with higher production efficiency, such as glycolysis, for facilitating the replication of SARS CoV 2 (44). Meanwhile, we also established that muscular disease, such as myositis, is associated with COVID-19. Previous studies have demonstrated that patients with dermatomyositis have three immunogenic linear epitopes with a high degree of sequence identity to the SARS-CoV-2 protein, so potential exposure to the coronavirus family may lead to the development of dermatomyositis (45). Effective Janus kinase (JAK) inhibitors for dermatomyositis, including tofacitinib, ruxolitinib, and baricitinib, may provide new directions for COVID-19 treatment.

In conclusion, the study provides new insights for the common pathogenesis of COVID-19 and atherosclerosis by looking for common transcriptional features. The DEGs identified by bioinformatics data analysis, including C1QA, C1QB, C1QC, CD163, SIGLEC1, APOE, MS4A4A, VSIG4, CCR1, and STAB1, may be therapeutic targets for the atherosclerosis caused by COVID-19. However, more wet lab-based studies are required to validate the impact of COVID-19 severity on atherosclerosis. Studies on the long-term effects of SARS-CoV-2 infection, the effect of persistent endothelial dysfunction on atherosclerosis, and the role of preventive therapy are also needed.

## Data availability statement

The datasets presented in this study can be found in online repositories. The names of the repository/repositories and accession number(s) can be found in the article/supplementary material.

## Author contributions

JZ performed the data analyses and wrote the manuscript. LZ helped perform the analysis with constructive discussions. Both authors approved the final version of the manuscript.

## Conflict of interest

The authors declare that the research was conducted in the absence of any commercial or financial relationships that could be construed as a potential conflict of interest.

## Publisher's note

All claims expressed in this article are solely those of the authors and do not necessarily represent those of their affiliated organizations, or those of the publisher, the editors and the reviewers. Any product that may be evaluated in this article, or claim that may be made by its manufacturer, is not guaranteed or endorsed by the publisher.
